# Bovine respiratory syncytial virus in experimentally exposed and rechallenged calves; viral shedding related to clinical signs and the potential for transmission

**DOI:** 10.1186/s12917-019-1911-z

**Published:** 2019-05-20

**Authors:** Thea Blystad Klem, Siri Kulberg Sjurseth, Ståle Sviland, Britt Gjerset, Mette Myrmel, Maria Stokstad

**Affiliations:** 1 Norwegian Veterinary Institute, P.O. Box 750 Sentrum, 0106 Oslo, Norway; 2 Department of Food Safety and Infection Biology, Norwegian University of Life Sciences, P.O. Box 8146 Dep, 0033 Oslo, Norway; 3 Department of Production Animal Clinical Sciences, Norwegian University of Life Sciences, P.O. Box 8146 Dep, 0033 Oslo, Norway

**Keywords:** BRSV, Experimental infection, Clinical signs, Virus shedding, Transmission potential, Biosecurity, RT-ddPCR, Virus isolation

## Abstract

**Background:**

Bovine respiratory syncytial virus (BRSV) is an important respiratory pathogen worldwide, detrimentally affecting the economy and animal welfare. To prevent and control BRSV infection, further knowledge on virus shedding and transmission potential in individual animals is required. This study aimed to detect viral RNA and infective virions during BRSV infection to evaluate duration of the transmission period and correlation with clinical signs of disease. The outcome of BRSV re-exposure on calves, their housing environment and effect of introduction of sentinel calves was also investigated.

A live animal experiment including 10 calves was conducted over 61 days. Initially, two calves were inoculated with a non-passaged BRSV field isolate. Two days later, six naïve calves (EG: Exposed group) were introduced for commingling and four weeks later, another two naïve calves (SG: Sentinel group) were introduced. Seven weeks after commingling, EG animals were re-inoculated. Clinical examination was performed daily. Nasal swabs were collected regularly and analysed for viral RNA by RT-ddPCR, while virus isolation was performed in cell culture. BRSV serology was performed with ELISA.

**Results:**

All the EG calves seroconverted and showed clinical signs of respiratory disease. Viral RNA was detected from days 1–27 after exposure, while the infective virus was isolated on day 6 and 13. On day 19, all animals were seropositive and virus could not be isolated. Total clinical score for respiratory signs corresponded well with the shedding of viral RNA. The SG animals, introduced 27 days after exposure, remained negative for BRSV RNA and stayed seronegative throughout the study. Inoculation of the EG calves seven weeks after primary infection did not lead to new shedding of viral RNA or clinical signs of disease.

**Conclusion:**

Viral RNA was detected in nasal swabs from the calves up to four weeks after exposure. The detection and amount of viral RNA corresponded well with the degree of respiratory signs. The calves were shedding infective virions for a considerable shorter period, and naïve calves introduced after four weeks were not infected. Infected calves were protected from reinfection for at least seven weeks. This knowledge is useful to prevent spread of BRSV.

## Background

Bovine respiratory syncytial virus (BRSV) is an important respiratory pathogen in cattle, detrimentally affecting the economy and animal welfare. The virus is distributed worldwide and is a major pathogen of the bovine respiratory disease complex [1, 2]. Viral respiratory infections are also of concern with regards to antibiotic resistance, as they predispose cattle to secondary bacterial infections that are commonly treated with antibiotics [3]. Bovine respiratory disease is traditionally handled with management measures, vaccination and metaphylactic antibiotic treatment [4]. Another possible strategy is to prevent inter-herd transmission of the main pathogens by increasing biosecurity measures at herd level. Because live animal transport is considered one of the main modes of BRSV transmission between herds [5, 6], proper mitigation must ensure that live animal transport be performed without compromising biosecurity. This requires knowledge on transmission risk associated with animal contact at different stages of infection. Knowledge of BRSV shedding related to clinical features would also be useful in order to assess the transmission risk of an infected herd without the use of viral diagnostic assays. For both of these areas, several knowledge gaps exists. Although way of infection may affect both viral shedding and clinical signs compared to naturally exposed animals, challenge studies are superior in the sense that aetiology and time of exposure is known and clinical features and virus excretion can be followed closely. Challenge studies, many of them aiming to evaluate the efficacy of vaccines [7–11] seldom last longer than one to two weeks. Grissett et al. [12] and Gershwin [13] concluded that shedding of BRSV begins on day three or four post-infection (p.i.) and usually lasts until day nine or ten. Grissett et al. [12] summarized that the median time to appearance, peak and resolution of clinical signs was 3, 6 and 12 days, respectively, based on information from 22 inoculation studies [7–11, 14–22]. As studies outlasting the acute phase of infection are lacking, it is not known how long an animal can transmit infectious viruses to other animals. Appearance of clinical signs is usually the only information available in the field, and finding a clinical parameter that indicates shedding of infectious BRSV would be valuable. The existence of chronic or persistent infections in individuals is likewise still unclear [23–26].

During the acute phase of a BRSV infection, immunological protection develops, but it is assumed to be short-lived [27]. This might enable early reinfection and new shedding of the infective virus, which complicates the risk assessment. A few BRSV studies have been performed to shed light on this. In a study by Kimman et al. [23] they reported a strong local IgA response in the respiratory tract, but no virus shedding, when calves were re-exposed 3–4 months after primary BRSV infection. Stott et al. [28] indicated, referring to their own unpublished results, that reinfection in calves and heifers may occur as early as three weeks post-infection. However, early reinfection with BRSV is not well-documented, and more precise knowledge of the occurrence is needed.

The existing literature on BRSV shedding and transmission is based on various laboratory methods, such as detection of viral RNA and culturing of the virus. Although resource-demanding, virus transmission studies are preferably performed using live animals in sentinel trials.

The aim of the present study was, therefore, to study basic features of BRSV infection in calves infected by exposure to BRSV-shedding calves. This was performed by:

1. Investigating the shedding of viral RNA and infective virions:
  - related to clinical outcome during the experimental period, lasting for two months
  - in calves rechallenged by inoculation seven weeks p.i.
2. Investigating whether the calves and their environment are not infectious to naïve in-contact calves four to nine weeks post-infection despite rechallenge with BRSV and mild stress induction.

## Methods

### Study design

A descriptive live animal experiment with a total of 10 calves was performed. The experimental units were groups of calves, and the total experimental period was 61 days. All the calves were euthanized by the end of the experiment. The groups were designated as Inoculated group (IG: calf I_1_ and I_2_), Exposed group (EG: calf E_1_-E_6_), and Sentinel group (SG: calf S_1_ and S_2_). The intervention for IG was inoculation with BRSV, while for EG it was exposure to IG from their acute stage until 26 days after challenge (D26) and inoculation with BRSV on D49. The intervention for SG was exposure to EG and their environment 27 days after first exposure to BRSV (D27). The read-out was presence of BRSV RNA, infective BRSV, anti-BRSV antibodies, clinical signs, weight gain and pathological lesions. Each single animal was used as its own control, mainly to detect whether the calves were clinically healthy, had an average weight gain and did not have antibodies against relevant respiratory viruses (BRSV, BCoV and BPIV3) before exposure to BRSV. The experiment was performed as an open-label trial, as blinding was not practically achievable.

**Fig. 1:**
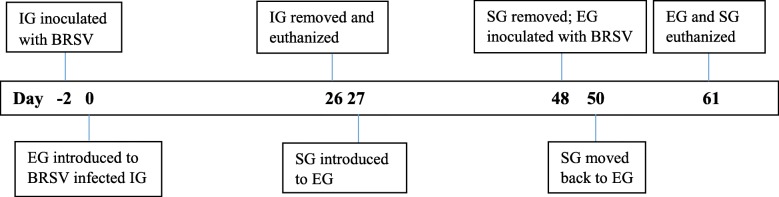
Timeline of interventions during the experimental period. Three groups of animals were included in the experiment: the Inoculated group (IG: calf I_1_ and I_2_), Exposed group (EG: calf E_1_-E_6_), and Sentinel group (SG: calf S_1_ and S_2_)

A timeline of the experimental period with an overview of interventions is provided in Fig. 1.

### Housing and husbandry

The experiment was conducted in the Laboratory Animal Unit at the Norwegian Veterinary Institute in Oslo, in a BSL2 facility consisting of three housing units with separate ventilation systems, and separate entrances with sluice rooms. In the sluice, the clothes and boots of the personnel were changed and hands were washed routinely, both before and after entrance to the calves. Each unit was provided with separate equipment for care and sampling and had one free-range housing pen with ad libitum water supply. The pens were bedded with sawdust and cleaned manually by a technician once a day. The pen where the IG was inoculated was 7 m^2^ and the two other pens were 8 m^2^ each. Roughage was fed ad libitum and a commercial concentrate formula was allotted twice a day. The allotment of concentrate started at 1 kg per day, gradually increasing to 2 kg within the first three weeks. From three weeks and throughout the study period, the ration was stable at 2 kg per day.

### Experimental animal arrangement

Ten BRSV seronegative, conventionally reared and weaned Norwegian Red calves aged 2–4 months were included. The two heifer and eight bull calves were obtained from three private Norwegian dairy farms situated in the southeastern part of Norway, in proximity to the BSL2 facility. The allocation of animals to experimental groups was random with the exception that calves of different age were mixed to avoid age bias. The first eight calves were initially housed in three different rooms. After the 11-day acclimatization period, the two IG calves (room 3), were inoculated with BRSV (D-2 of the experiment). Two days later (D0), I_1_ and I_2_ were moved to room 1 and 2, respectively, each holding three EG calves. The EG calves were housed together with the IG calves until D26, when I_1_ and I_2_ were euthanized. On D27, S_1_ and S_2_ were brought in to rooms 1 and 2, respectively, where the EG calves were housed. On D48, the two SG calves were moved to a separate room (room 3). At the same time, the EG calves were rearranged to induce mild stress: Two of three calves in each room were moved to the other room, and rechallenged by inoculation with BRSV. On D50, S_1_ and S_2_ were moved back to room 1 and 2, respectively. On D61, the remaining eight calves were euthanized.

### Inoculum and inoculation procedure

Prior to inoculation, a BRSV field isolate O4-4B/N/11 earlier described by Klem et al. [29] was centrifuged at 1200 x g and filtered through a 0.45 μm filter. To exclude the possibility of bacterial contamination the filtrate was seeded out on blood agar plates and cultured for 24 h at 37 °C. The filtrate was also tested by RT-PCR for the presence of BRSV, bovine parainfluenza-3 virus (BPIV-3) [29] and bovine corona virus (BCoV) (in-house method, Norwegian Veterinary Institute, Oslo, Norway). The filtrate was positive for BRSV with a Ct-value of 21.4 and negative for BCoV, BPIV-3 and bacteria. The inoculum was prepared by diluting the filtrate 1:5 with sterile NaCl.

The inoculation procedure was performed on non-sedated calves via manual restraint. The head was kept in a horizontal position while 5 ml inoculum was nebulized intranasally by use of a perforated plastic tube with a diameter of 1.57 mm and an inner channel of 1.14 mm. The tube was inserted 15 cm into the left nostril. After nebulization, the left nostril was closed by hand to avoid outflow of the inoculum.

### Treatment procedures

Moderately to severely depressed calves with a high respiratory rate (≥ 65/min) and temperature ≥ 40.0 °C were treated with 0.5 mg/kg meloxicam for pain relief and anti-inflammatory effect. When bacterial pneumonia was suspected, 20 mg/kg procain benzylpenicillin was administered IM once daily for five days. Euthanasia was performed by stunning with a captive bolt, followed by bleed-out.

### Clinical observations

Qualified personnel monitored the calves morning and evening, and more frequently if deemed necessary. A veterinarian examined all calves on a daily basis until D10, and three to four times a week thereafter throughout the experimental period. When the general health condition of the calves was negatively affected, the frequency of examination was increased. A clinical scoring system was developed, modified after Hägglund et al. [30], and is presented in Table 1. An overall clinical score was calculated by summing up the separate scores from the clinical registrations. The peak outbreak was defined as the day with the highest number of clinically affected calves.

**Table 1:** Clinical scoring system. The score from each category was added to give a daily clinical score for each of the calves in the experiment

Score	Respiratory rate (breaths/min)	Rectal temperature (°C)	Cough	Nasal discharge	Lung auscultation	Demeanor
0	≤ 49	≤ 39,5	No cough observed	Normal	No abnormal sounds	Bright, alert
1	50–54	39,6–39,9	Sporadic cough	Serous or mucous	Wheezing sounds	Mildly depressed
2	55–64	40–40,4	More than one sporadic cough every 10 min of observation	Mucopurulent or purulent	–	Moderately depressed
3	65–74	40,5–40,9	–	–	–	Severely depressed
4	75–85	> 40,9	–	–	–	–

Mild score: clinical score > 2 on 3 consecutive days

Moderate score: clinical score ≥ 4 on 3 days or more

Severe score: clinical score ≥ 9

### Collection of biomaterial

Collection of blood samples and nasal swabs was performed three to four times a week from each animal. Nasal swabs (ESwab™ Copan, Brescia, Italy) for virus detection were placed approximately five cm into the right and the left nostril, alternately. The swab was pressed gently against the nasal septum and rolled up and down four times.

All swabs were kept on ice and stored at − 80 °C within one hour, until analysis.

Blood was collected from the external jugular vein in plain tubes. Sera were stored at − 20 °C until analysis.

### Quantification of BRSV RNA (RT-ddPCR)

All the nasal swabs collected during the experimental period were analysed. RNA was extracted from 200 μl of the swab transport medium using the automated NucliSens easyMAG protocol (Biomérieux, Marcy l’Etoile, France), according to the manufacturer’s instructions. Quantification of BRSV genomes was conducted in duplicate, with Bio-Rad’s QX200 ddPCR System (droplet digital PCR). Each run included a positive (RNA from the nostril of a BRSV-positive trial calf) and negative control (water). Droplet generation and transfer of droplets were as described by the manufacturer. The One-Step RT-ddPCR Advanced Kit for Probes (BioRad, CA, USA) and 2 μl RNA were used. The sequence of primers and probe (5′ FAM and BHQ1 as quencher) was as described by M. Boxus et al., targeting a 123 bp region of the BRSV N gene [31]. Primers and probe concentrations were as recommended by the kit manufacturer and with the following cycling conditions: 50 °C for 60 min, 95 °C for 10 min and 40 cycles of 95 °C for 30 s and 60 °C for 1 min. The ramp rate was set to 2 °C/second. Data processing and absolute quantification of BRSV genomes per μl RNA was performed with QuantaSoft Version 1.7 (BioRad, CA, USA).

Positive nasal swabs from the calves were subjected to Kaplan-Meier survival analysis in Stata (Stata SE/ 14, Stata Corp., College Station, TX, USA). The function shows the cumulative survival, i.e. shedding of BRSV RNA over time, which decreases as nasal swabs from the calves turns BRSV RNA negative. As the exact time-point the nasal swab of a calf turned negative was unknown, the mid-point between the last positive and the following negative sample was used in the analysis.

### BRSV isolation

Based on RT-ddPCR results, nasal swabs were selected for virus isolation at three different time points from all calves in group EG: 1) Early—D6, the second day that all calves tested positive for viral RNA; 2) Middle—D13, all calves were still positive; and 3) Late—D19, the first day where viral RNA was no longer detected in all EG calves (five of the six were positive).

Virus isolation was performed by inoculating foetal bovine turbinate (FBT) cells propagated in Eagle’s minimal essential medium (EMEM, Belgium) in 96 well plates in duplicate samples of 50 μl of 0,45 μm filtered nasal swab medium for 30 min. After adding medium with 2% FCS, negative replication control cells were harvested, while the parallel samples were incubated at 37 °C, in 5% CO_2_. The cells were observed for cytopathogenic effect (CPE) and passaged after 7 days of incubation. Presence of CPE was further confirmed by a direct immunofluorescence test using FITC Moab a-BRSV (Bio-X Diagnostics, Rochefort, Belgium) and supernatants were tested by the BRSV RT-ddPCR described.

### Antibody detection

Indirect ELISAs for the detection of serum antibodies specific to BRSV (SVANOVIR® BRSV-Ab), BPIV-3 (SVANOVIR® PIV3-Ab) and BCoV (SVANOVIR® BCV-Ab) (all from Svanova Biotech AB, Uppsala, Sweden) were performed according to the manufacturer’s instructions. Sera collected from all calves every second day from D-2 to D60 were analysed for BRSV antibodies. In addition, sera were analysed for detection of antibodies against BCoV and BPIV-3 at specific time points: For IG on D-2 and D26, for EG on D26, D47 and D60, and for SG on D27, D47 and D60.

### Pathological examination

Standard post-mortem examinations were performed on infected animals (IG and EG), including macro- and micro-pathological examinations of the respiratory tissue. Lung tissue was prepared and cultured for the presence of bacteria and sections were stained with haematoxylin-eosin for histological investigation.

### Weight gain

The calves were weighed two to three times per week throughout the experimental period. Average daily weight gain was calculated for the infected animals (IG and EG) by dividing total weight gain by the number of days between each weighing. The median of the average daily weight gain (all calves) were calculated and the minimum (min) and maximum (max) median values were registered for the acclimatization period (D-11–D0), the peak infection period (D1–D15) and the restitution period (D16–D57).

## Results

### Clinical outcome

All IG and EG calves showed signs of respiratory disease, varying from mild to severe (Table 2). In IG, the first clinical sign was observed three days after inoculation (D1), when one calf had sparse serous nasal discharge. Four days later, both inoculated animals showed mild respiratory signs with mild depression, normal to seromucous nasal discharge and a sporadic cough. Nine days after inoculation (D7), the total clinical score was at its highest for the IG calves, and the median of the total clinical score value was 4.0. On D1, the first clinical sign appeared in three of the six EG calves as they showed mild signs of respiratory disease, including a sporadic cough, mild depression and/or sparse serous to mildly opaque nasal discharge. Calves E_5_ and E_6_ started to show severe signs of respiratory disease on D10 and D15, respectively, which qualified for medical treatment with penicillin and anti-inflammatory drugs. The peak outbreak was on D11 with all EG animals showing sign of disease and the highest total clinical score with a median value of 2.5 (min 1, max 13). During the trial, five out of six of the EG calves had a temperature equal to or higher than 40.0 °C. The highest median rectal temperature of the calves in EG occurred on D12 and D15, with the median temperature being 39.6 °C both days (D12: min 38.6 °C, max 39.7 °C, D15: min 38.9 °C max 40.6 °C). The SG calves did not develop respiratory disease.

**Table 2:** Key clinical signs of respiratory disease after experimental infection with BRSV

Animal group	Calf no.	Peak rt. °C	Number of days with				Peak clinical score	Days with clinical score ≥ 4
			depression	respiratory rate ≥ 65	nasal discharge	cough with clinical score ≥ 2		
Inoculated	I_1_	40.1	4	0	10	8	7	2
	I_2_	39.6	2	0	2	1	5	3
Exposed	E_1_	40.6	4	0	7	3	6	3
	E_2_	40.0	7	0	5	6	6	6
	E_3_	40.4	5	0	5	2	6	3
	E_4_	39.2	2	0	9	3	4	2
	E_5_	41.2	4	6	9	2	10	4
	E_6_	40.3	4	2	2	4	9	4

**Fig. 2:**
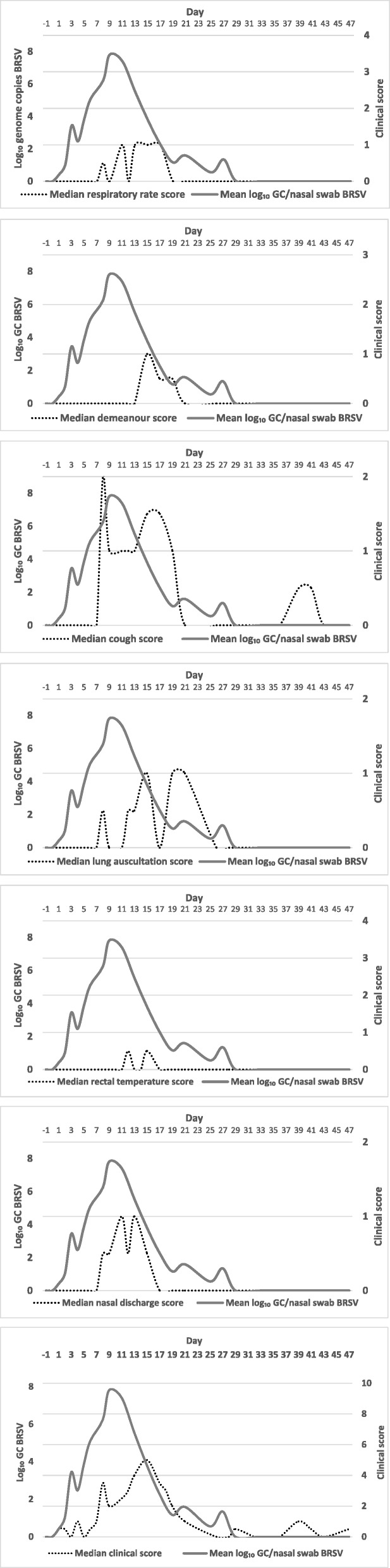
Mean number of genome copies of bovine respiratory syncytial virus (BRSV) related to median clinical score of infection. Day = days post-exposure to BRSV, GC = genome copies

The association of the BRSV RT-ddPCR results versus scores of clinical parameters and total clinical score of the EG calves is shown in Fig. 2.

### Viral RNA in nasal swabs

**Fig. 3:**
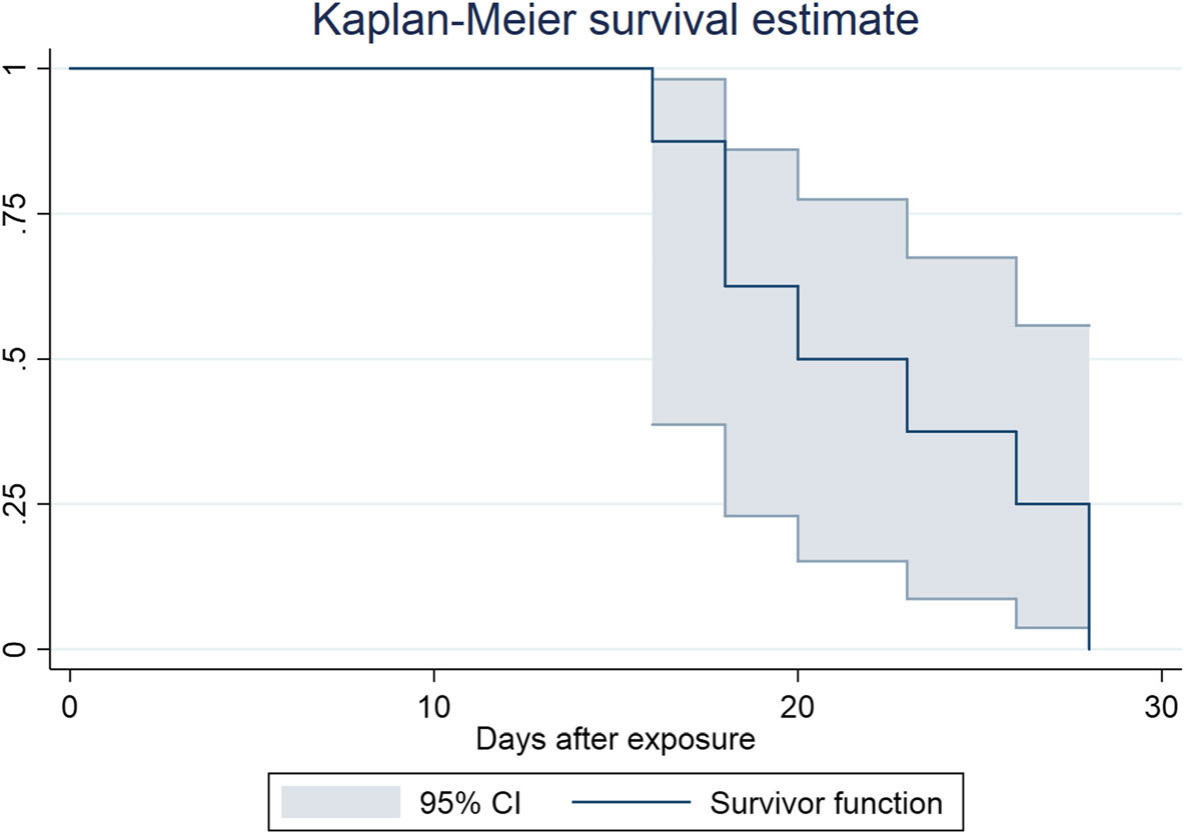
Kaplan-Meier survival function for BRSV RNA in nasal swabs from infected calves

BRSV RNA was detected in nasal swabs from all IG and EG calves after the first challenge, and quantitative RNA values are given in Table 3. In IG, the first positive sample was detected two days after inoculation (D0). In EG, RNA was first detected on D1, and all animals were positive by D5. The peak was on D9. One IG calf was positive until D17, while two EG calves were still positive on D27. From D29, nasal swabs from all calves were negative. The respective log_10_ numbers of genome copies are found in Table 3. The results of the Kaplan-Meier-survival analysis of viral RNA in nasal swabs from the calves are provided in Fig. 3. According to the analysis, the estimated longest persistence of viral RNA was until 28 days after exposure.

**Table 3:** Log_10_ numbers of genome copies of BRSV per swab in calves of inoculated group (IG, calf I_1_ and I_2_) and exposed group (EG, calf E_1_–E_6_) from first day of exposure of IG to EG to the last day where nasal swabs positive for viral RNA were detected with ddPCR (D27)

Day post- exposure EG	Log_10_ genome copies BRSV/swab in IG and EG calves							
	I_1_	I_2_	E_1_	E_2_	E_3_	E_4_	E_5_	E_6_
0	3.0	–	–	–	–	–	–	–
1	5.2	–	–	–	–	–	2.5	–
2	7.5	7.5	–	–	–	2.8	–	3.3
3	7.9	–	6.0	–	2.2	4.7	4.3	3.5
4	8.0	7.4	2.7	–	4.3	–	3.8	4.0
5	8.6	7.3	3.1	3.3	2.5	3.6	6.4	4.2
6	7.7	7.3	3.7	4.8	2.9	5.3	7.6	6.3
8	4.6	4.3	4.9	5.6	3.4	7.5	8.1	8.0
9	5.1	4.8	7.4	6.8	7.3	8.5	8.7	8.1
11	4.4	4.1	7.8	7.8	8.2	7.4	6.4	6.8
13	4.0	4.1	6.9	6.9	5.9	3.9	4.9	4.7
15	3.1	3.4	3.5	3.7	3.4	3.6	5.6	3.0
17	–	3.1	–	4.6	–	–	4.8	4.2
19	–	–	–	–	–	3.2	3.8	–
21	–	–	–	–	–	4.6	–	5.0
25	–	–	–	–	3.3	–	–	–
27	–	–	–	–	–	3.3	2.5	–

The SG calves were negative for viral RNA throughout the study period and none of the EG calves were positive following the rechallenge.

### Detection of infective viruses in nasal swabs

Cytopathogenic effect and positive immunostaining were observed in cells incubated with swab material from all six EG calves collected on D6 and D13. No clear CPE or immunostaining were caused by samples collected on D19. A high difference in supernatant viral RNA titer was found by RT-ddPCR between negative replication control and incubated samples from D6 and D13 (range: 2.9–7.6 and 2.5–5.3 log_10_ GC per swab, respectively). Samples collected on D19 did not show any rise in supernatant viral RNA titer after two passages in FBT cells, which indicates no infective BRSV.

### Serology

**Fig. 4:**
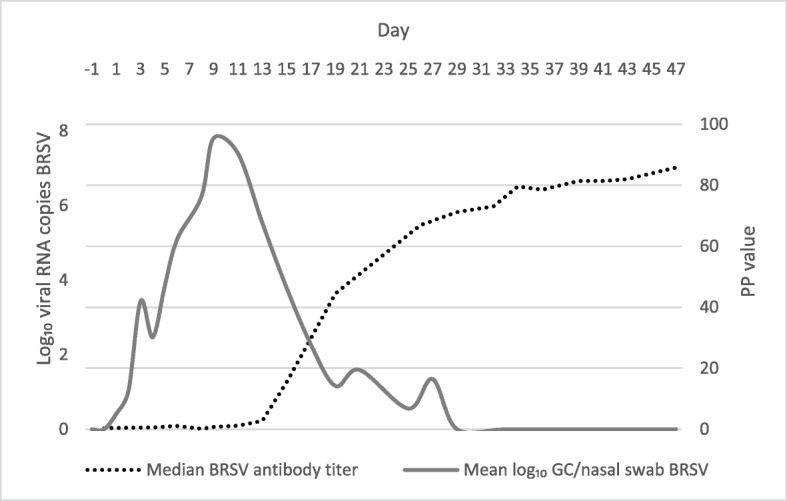
Mean number of genome copies of bovine respiratory syncytial virus (BRSV) related to median antibody titre. Day = days post-exposure to BRSV, GC = genome copies, PP = percent positivity

All calves tested negative for antibodies against BRSV before inoculation/exposure. In IG, one calf seroconverted on D11, and both calves were positive on D13. In EG calves, seroconversion was first detected on D13, and all calves had seroconverted by D19. SG calves remained seronegative to BRSV throughout the study period. In Fig. 4, the corresponding median values of EG BRSV antibody titer and RNA quantities are presented. No animals seroconverted towards BCoV or BPIV-3.

### Pathological examination

All IG and EG animals had mild pathological lesions in the respiratory tract, with bronchointerstitial pneumonia, ranging from mild to moderate. Five out of eight calves had a mucopurulent bronchitis and/or bronchiolitis, six had a mild bronchiolitis obliterans, seven had mild to pronounced atelectasis and one calf had pleuritis. All the calves had mild to pronounced bronchus-associated lymphoid tissue hyperplasia and mild to moderate reactive hyperplasia of the lymphatic tissue of nasopharynx and the regional lymph nodes of the lungs. Bacterial culture from lung tissue showed no growth, except from one calf, where sparse occurrence of *Trueperella pyogenes* was identified.

### Average daily weight gain

During the acclimatization period (D − 11 to D0), the median of the average daily weight gain (all calves) was 991 g (min 878 g, max 1500 g). In the peak infection period (D1–D15), it was reduced to 583 g (min 117 g, max 1000 g), while increasing to 901 g (min – 1350, max 1850 g) in the restitution period (D16–D57).

## Discussion

The present study aimed to assess the transmission potential of BRSV from infected calves and their housing environment in an experimental setting. Only ten animals were included in the study. Although not ideal, the chosen study design is common in experimental infection studies in large animals [12]. This in contrast to other type of studies with traditional laboratory animals or more epidemical studies, e.g. natural infections in the field. There are several challenges related to the large size of the animals like the accessibility to experimental facilities of acceptable size and biosecurity level. It is also an economical aspect due to the high costs of housing in experimental facilities and to the cost of purchasing calves. The ethical aspect according to number of animals used is always of concern. Consequently, the present study is performed as a descriptive experimental infections study.

The experimental setting allowed controlled conditions and control of time of exposure to BRSV, which is difficult to achieve under field conditions. Natural conditions were mimicked concerning host species, age, breed, feeding and social interaction with other calves. Several environmental factors for the calves are identified as risk factors of respiratory disease. Among these are low temperatures combined with wetness of the animals, dust, herd size, transport, shared housing between calves and adult cattle, large differences in age and commingling of many calves in the same pen [32, 33]. In the present experiment, most of these factors were not advisable or possible to implement, but a few mild stressors were introduced, such as moving animals and commingling with other calves (i.e., rearrangement during the experiment). The bedding was dry, leaving the animal rooms dustier than in an average herd. Although severe respiratory disease was observed, clinical symptoms might have been even more severe if the virus had been introduced to a regular cattle herd. A non-passaged BRSV strain obtained from a field outbreak of respiratory disease was used for inoculation, in order to avoid changes in virulence due to cultivation in cell lines. For the main calf group (EG), natural exposure to virus-shedding calves was used to mimic field conditions. Experimental studies usually include control groups where naïve animals are kept in the same environment. However, due to biosecurity measures and room capacity, the present housing facilities did not allow for this. Therefore, each calf functioned as its own control, by using the period prior to the challenge as a control period.

The long experimental period allowed the study of the presence of nasal mucosa-associated viral RNA for an extended time period, which revealed that BRSV was present for up to four weeks, starting on D1–D5. Compared to results of the group of calves investigated in the present study, previous studies have concluded that viral shedding usually begins later, and lasts for a shorter period [7–11, 13–22]. Other studies have claimed that BRSV replication in the upper respiratory tract only occurs in an early stage of infection [34, 35], which has led to the conclusion that sampling of bronchoalveolar lavage and lung tissue is necessary for diagnostic purposes in later stages of the disease. The present results indicate that diagnostic sampling using nasal swabs is suitable for up to one month after exposure to BRSV. This is in line with another study by Klem et al. [29], and enables easier, cheaper and more frequent testing of animals at risk.

Although viral RNA was found in nasal swabs for up to four weeks, cell culture propagation of BRSV from nasal swabs indicated that the shedding period of infectious virus amounts is considerably shorter, approx. 13–18 days, indicating a shorter period of actual transmission risk. The two SG calves remained uninfected after commingling with EG calves and their environment four weeks after EG calves were exposed to BRSV. The number of calves was unfortunately too low to conclude if this would be valid in a real herd with a larger number of animals, Nevertheless, it would be interesting to investigate this further in a study with more animals included and a sequential study design. In a recent experimental infection study on BCoV [36], a similar pattern was observed; RNA from BCoV was detected for approximately a month after infection, but the infectivity of the virus was found to be considerably shorter, lasting for up to 18 days. These results suggest that the same strategies can be applied to prevent inter-herd transmission for both bovine respiratory disease complex pathogens.

Some differences were found in the viral shedding patterns of EG and IG calves, which might reflect differences between infection caused by natural exposure to the virus and the more artificial inoculation procedure—e.g., the time from exposure until the detection of viral RNA in the nasal swabs was shorter in EG calves than the time from inoculation to viral shedding in IG calves. The virus in the nasal swabs might reflect the presence of the inhaled virus, but no active BRSV infection. Bovine respiratory syncytial virus is a labile, enveloped virus and freezing and thawing can detrimentally affect infectivity [2, 37, 38]. As the amount of infective virus was unknown in the inoculate, it may have contained less infective virus than the amount the EG calves were exposed to. This might explain why inoculation led to a longer incubation period and less severe infection in IG calves. In addition, viral RNA was detected for a longer period of time in the EG calves. The late outbreak peak of clinical signs of the EG compared to earlier reports from experimental infections [12] might have been caused by a more prolonged process in a group of animals naturally exposed to BRSV. This adds to the view that studies based on natural exposure are more valid than inoculation studies.

Clinically, the infection resembled a naturally occurring BRSV infection, with all infected calves showing respiratory signs but with individual differences in severity. Comparison of the median values of the total clinical score and the BRSV RNA titer showed a clear conformity between the two parameters during the course of the trial. In general, the detection of replicating viral RNA occurred a few days before the onset of clinical signs. Rise in temperature and coughing is commonly used as a measure to detect respiratory disease in the field. In the present experiment, these parameters increased during the same time period as the number of BRSV genome copies raised. However, the coughing continued intermittently for a much longer period than viral RNA was detected. The median values of the rectal temperature score was only slightly elevated. Increased respiratory rate and nasal discharge appeared to be the clinical parameter corresponding best with the detection of viral RNA for the included calves. The clinical signs also corresponded well with the periods when an active BRSV infection was detected. After approximately three weeks, no infective virus could be isolated and most clinical signs were very mild or no longer present.

The pathological examination showed that both IG and EG calves had mild pathological lesions in the respiratory tract. This was unexpected, as the calves were no longer clinically ill at the time of euthanasia. Nevertheless, the low average daily weight gain in the peak infection period and the failure to regain normal growth during the recovery period are in agreement with previous findings indicating a negative effect on growth and production, months after apparent recovery [39].

During infection, the calves showed clinical signs and pathological changes normally associated with BRSV infection in calves without co-infections. However, infections with BRSV are known to predispose cattle to secondary bacterial infections, and despite the fact that bacterial examinations of lung tissue post mortem were negative in all calves but one (who had sparse numbers of *Trueperella pyogenes*), it is not possible to completely rule out the possibility of co-infections—especially considering the fact that, during the experiment, two of the calves were medically treated with penicillin. BRSV is also known to occur in co-infection with other viruses or with *Mycoplasma spp*. In the present study, none of the animals were infected with BCoV or BPIV-3, and the Norwegian cattle population is currently free from many of the internationally recognized agents of bovine respiratory disease; for example, bovine herpes virus type 1 [40], bovine viral diarrhoea virus [41] and *Mycoplasma bovis* have never been detected [32].

Calves in the present study were not reinfected by inoculation seven weeks after the primary infection, indicating that protective immunity is longer than what has previously been suggested [23, 28]. The sentinel animals remained seronegative after contact with the EG animals one month after the onset of infection, and the seroconversion in the EG calves after about two to three weeks, coincided with the period when the active infection had been cleared and the virus no longer seemed to be infective in the cell culture.

Taken together, protective immunity seemed to correspond with the detection of serum antibodies, and to last for at least 7 weeks.

The median clinical score, mean viral RNA detection and virus infectivity coincided and were strongly reduced about three weeks after exposure to BRSV. The negative results in the sentinel study further support the argument that BRSV infected animals and their housing environment pose a lower risk after three to four weeks. Combining this knowledge with the registration of clinical signs of respiratory disease, with an emphasis on the occurrence of increased respiratory rate, could be useful information when implementing preventive biosecurity measures—such as periods of quarantine—to avoid spreading the virus. However, an experimental setting with relatively few animals of the same age will differ from the transmission dynamic in a regular herd. One of the challenges in a conventional herd setting is to know at which time point the virus has circulated throughout the whole herd. Since animals may develop subclinical infection, the time point when the last animals clear the infection will often be unknown. Despite the efforts in this experiment to mimic natural conditions, these factors must be considered when the results are applied in a herd.

## Conclusions

In this study, viral BRSV RNA was detected in nasal swabs from calves up to 27 days after exposure to the virus. The period when infective viruses were found was shorter: between 13 and 19 days. The total clinical score, the increase in the respiratory rate and nasal discharge corresponded best with the shedding of viral RNA. Contact between EG calves and naïve calves four weeks after exposure did not result in infection. EG calves were protected from reinfection seven weeks after initial exposure. This adds to our basic understanding of transmission patterns of BRSV, within- and between-herds, and can provide support for preventive action when used to design inter-herd biosecurity measures.

## Abbreviations

BCoV: Bovine corona virus
BPIV-3: Bovine parainfluenza-3 virus
BRSV: Bovine respiratory syncytial virus
CPE: Cytopathogenic effect
D: Days after challenge
EG: Exposed group
FBT cells: Foetal bovine turbinate cells
GC: Genome copies
IG: Inoculated group
P.i.: Post infection
PP: Percent positivity
RT-ddPCR: Reverse transcription droplet digital polymerase chain reaction
SD: Standard deviation
SG: Sentinel group
